# Ipsilesional Impairments of Visual Awareness After Right-Hemispheric Stroke

**DOI:** 10.3389/fpsyg.2019.00697

**Published:** 2019-04-09

**Authors:** Mario Bonato, Zaira Romeo, Elvio Blini, Marco Pitteri, Eugenia Durgoni, Laura Passarini, Francesca Meneghello, Marco Zorzi

**Affiliations:** ^1^Department of General Psychology and Padova Neuroscience Center, University of Padua, Padua, Italy; ^2^IRCCS San Camillo Hospital, Venice, Italy; ^3^Integrative Multisensory Perception Action and Cognition Team, University Claude Bernard of Lyon, Lyon, France; ^4^Neurology Section, Department of Neurosciences, Biomedicine and Movement Sciences, University of Verona, Verona, Italy

**Keywords:** spatial processing, spatial awareness, consciousness, stroke, hemianopia, neglect, ipsilesional

## Abstract

Unilateral brain damage following stroke frequently hampers the processing of contralesional space. Whether and how it also affects the processing of stimuli appearing on the same side of the lesion is still poorly understood. Three main alternative hypotheses have been proposed, namely that ipsilesional processing is functionally (i) hyperefficient, (ii) impaired, or (iii) spared. Here, we investigated ipsilesional space awareness through a computerized paradigm that exploits a manipulation of concurrent information processing demands (i.e., multitasking). Twelve chronic right-hemisphere stroke patients with a total lack of awareness for the contralesional side of space were administered a task that required the spatial monitoring of two locations within the ipsilesional hemispace. Targets were presented immediately to the right of a central fixation point (3° eccentricity), or farther to the right toward the screen edge (17° eccentricity), or on both locations. Response to target position occurred either in isolation or while performing a concurrent visual or auditory task. Results showed that most errors occurred when two targets were simultaneously presented and patients were faced with additional task demands (in the visual or auditory modalities). In the context of concurrent visual load, ipsilesional targets presented at the rightmost location were omitted more frequently than those presented closer to fixation. This pattern qualifies ipsilesional processing in right-hemisphere stroke patients as functionally impaired, arguing against the notion of ipsilesional hyperperformance, especially when under visual load.

## Introduction

Lesions of the right hemisphere often result in visuospatial deficits, such as the widely known hemispatial neglect and extinction. The pathognomonic clinical feature common to most of these disorders consists in impaired conscious processing for stimuli appearing in the side of space opposite to the damaged hemisphere (Driver and Vuilleumier, 2001; Corbetta and Shulman, 2011; Bartolomeo et al., 2012; Vuilleumier, 2013; Cubelli, 2017). Within the context of impairment of contralesional space processing, whether processing of ipsilesional space is intact, or just comparatively less affected, is a question that has not been often addressed. It is known that the presence and the severity of contralesional space impairment changes according to a wealth of factors including, for instance, the type of test (e.g., cancellation tasks vs. line bisection tasks, see Ferber and Karnath (2001), the level of motor involvement, and nature of the spatial domain investigated (e.g., peripersonal vs. extrapersonal space (Halligan and Marshall, 1991). On the top of that, mounting evidence suggests that the presence and severity of contralesional awareness deficits is strongly modulated by task demands. For instance, when multiple spatial locations are to be processed, an increased attentional engagement enlarges the neglected portion of contralesional space (Russell et al., 2004; Sarri et al., 2009). Moreover, when target position is kept constant, higher task demands result in increased omission rates (Bonato, 2012). Whether the same phenomena can be found within the ipsilesional side of space is largely unknown. Still, if we assume that neglect is task-dependent, the extent of space that is affected – and therefore the relationship between contra- and ipsilesional disorders – has to be considered direct. Let us consider, for instance, the performance of a neglect patient in a typical cancellation task: if the extent of the neglected space depends on task difficulty, a very difficult task might result in omissions extending from the contralesional toward the less lateralized portions of the ipsilesional side of space.

The dependency of contralesional space deficits on task difficulty clearly depicts neglect as a continuous disorder, not only when considering the extent of space neglected, but also when, more importantly, a diagnosis has to be made. Many patients with non-pathological scores might, in fact, simply present subclinical impairments which go undetected by standard methods (Pitteri et al., 2018). Similarly, patients with minor deficits might present severe patterns of omissions when tested with other methods. In order to better address the task-dependency issue, Bonato et al. (2010) devised a computer-based task with constant stimuli and varying attentional demands. In their approach, the detection rate of briefly presented, lateralized targets is compared across different conditions, allowing the patient either to focus on target stimuli only or requiring them to process additional, visual/auditory concurrently-presented stimuli (dual-tasking). Chronic stroke patients, under these more challenging conditions, show deficits for the contralesional space which are much more severe than those detected by standard, paper-and-pencil neuropsychological evaluation. Across a number of studies (Bonato et al., 2010) interpreted the emergence of contralesional omissions under multitasking as due to the impossibility to compensate for a spatial deficit which was present in a latent form. This approach also allowed us to characterize two possible core neglect features – namely its low frequency after left-hemisphere damage (Blini et al., 2016), and its putative stabilization in the chronic phases (Bonato, 2015) – as being task-dependent and, therefore, ascribed to the low sensitivity of standard clinical assessment methods. It should also be noted that brain damage can directly hamper contralesional visual perception by causing a pure visual deficit (i.e., homonymous hemianopia), which very often cannot be easily disentangled from severe neglect (Müller-Oehring et al., 2003).

In the present investigation, we capitalize on the sensitivity of the previously described multitasking approach (Bonato et al., 2010; Blini et al., 2016) to test how resilient to increased attentional demands is the “seemingly unimpaired” awareness for ipsilesional targets in right-hemisphere stroke patients. In the following sections we discuss three specific hypotheses about the functionality of ipsilesional space processing: the first posits that ipsilesional attention is enhanced, as suggested by the fact that it is strongly attracted by items appearing in the ipsilesional side of space; the second posits that performance in the ipsilesional side of space is impaired; the third simply assumes that ipsilesional space processing is unimpaired, at least with respect to its more lateralized sectors. Note that the first two hypotheses are not to be considered alternative, but rather complementary in considering ipsilateral processing as influenced by brain damage. The third hypothesis, instead, predicts that processing of (the most) ipsilesional spatial positions is not affected by brain damage.

### Ipsilesional Attraction

A first hypothesis is that patients with right hemisphere damage (RHD) might present a “magnetic” attraction toward ipsilesional stimuli (Gainotti et al., 1991). A landmark study (Mark et al., 1988) showed that the neglected side of space reduces when (ipsilesional) items are progressively erased after detection. This would relate the imbalance between neglected/non-neglected spatial extent to the presence of ipsilesional, non-neglected stimuli attracting attention. The study by Natale et al. (2007) showed that RHD patients with left neglect may be even faster than healthy controls in performing saccades toward ipsilesional targets. This, however, occurred only for slightly lateralized locations (within an off-centered ipsilesional sector of about 10°). When discussing about ipsilesional attraction, a different yet closely related phenomenon is the so-called disengagement deficit (for review see Losier and Klein, 2001), namely the specific difficulty in (re)orienting attention toward the contralesional space after having been (invalidly) cued toward the ipsilesional space. This bias is typically assessed using a Posner cueing task (Posner, 1980) and it strongly correlates with clinical indices of neglect (Morrow and Ratcliff, 1988). Further evidence often considered as supporting the ipsilesional ‘hyperprocessing’ hypothesis comes from extinction at double simultaneous stimulation, whereby ipsilesional targets are strongly prioritized, and hamper the report of simultaneously-presented contralesional ones (Vossel et al., 2011). However, the concurrent presence of ipsilesional attentional capture and contralesional omissions makes it difficult to disentangle the hyper- from the hypo-attentional component. In short, a number of heterogeneous proposals suggested that, at least in specific contexts, the processing of items appearing within the ipsilesional space appears to be facilitated.

### Deficient Ipsilesional Processing

A second possibility, thoroughly reviewed and tested by Chokron et al. (2018), is that ipsilesional processing should be more appropriately considered as impaired. At odds with the idea of ipsilesional facilitation, Chokron et al. (2018) reported that left neglect patients often have difficulties when responding to right-sided stimuli. According to this hypothesis, patients’ ipsilesional slowing would be strictly related to the severity of left neglect and would not – or at least not directly – reflect unspecific impairments (Bartolomeo and Chokron, 1999; Bartolomeo et al., 1999). As reported by Chokron et al. (2018), it is also possible to conceive the rightward attentional bias in left neglect patients as a paradoxical effect depending on task difficulty, which would thus manifest itself as ranging between facilitation for simple tasks and deficient performance in more complex ones (see also Bartolomeo and Chokron, 2000). According to this view, the seemingly hyperefficient ipsilesional space processing would be the consequence of a defective, and not enhanced, attention. The nature of this deficit has been attributed either to an unspecific loss of attentional capacity (see proposals by Robertson and Frasca, 1992; Robertson et al., 1998) or to a more specific selective attention impairment in filtering/prioritizing information (Snow and Mattingley, 2006).

Consistently with the idea of a left-to-right gradient in omissions, classical studies by Marshall and collaborators (Marshall and Halligan, 1989; Halligan et al., 1992), demonstrated that the modulation of neglect upon spatial processing is not dichotomous but continuous (see also Butler et al., 2004) and can extend, for some patients and tasks, to the ipsilesional space. The extent of space neglected by every single patient is strictly task-dependent (Sarri et al., 2009). At group level, a very clear spatial gradient is always present in cancellation tasks, whereby the detection of the more ipsilesional items is spared even in the most severe neglect patients. Evidence for a gradient has been extended to computer-based tasks by Smania et al. (1998). They reported that patients with RHD damage and left neglect omitted not only most of the contralesional targets, but also a significant number of those ipsilesionally presented immediately on the right of the fixation point. Interestingly, any “strong gradient” view suggests that the most ipsilesional spatial positions remain unaffected (see next paragraph).

### Normal Ipsilesional Space Processing

The third hypothesis is that performance in the ipsilesional side of space might be fully, or at least relatively, unimpaired. Attentional deficits after RHD follow a left-to-right gradient (Behrmann et al., 1997). Therefore, as previously stated, considering performance for ipsilesional targets as unimpaired does not seem necessarily inappropriate from a theoretical perspective. The assumption, usually implicit, is that ipsilesional deficits are absent or negligible, and this would allow ipsilesional performance to be taken as individual baseline for each patient. All in all, this is a very common assumption about neglect patients’ ipsilesional performance, at least when considering the most ipsilesional space sectors. Nevertheless, the observation of errorless performance for the most lateralized ipsilesional locations might depend on ceiling effects. Recently Machner et al. (2018) showed that the most severe neglect patients they tested were slower than controls in detecting ipsilesional targets in a Posner detection task, while in a search task they processed the most ipsilesional targets with the same accuracy as healthy controls (i.e., almost errorless).

In the present study we exploited the manipulation of concurrent information processing demands (i.e., multitasking) to investigate whether visual awareness for targets appearing within the ipsilesional side of space is hampered by RHD. Assessing the effect of multitasking can inform the above mentioned debate about the status of ipsilesional space processing in stroke patients.

## Materials and Methods

### Participants

Twelve stroke patients with right hemisphere damage (RHD) took part in the study. All patients were admitted to the San Camillo Neurorehabilitation Hospital (Lido di Venezia, Italy) to undergo motor and cognitive rehabilitation programs. All patients were in the sub-acute to chronic phase (minimum time from onset: 63 days, see Table 1). Seven healthy participants were also included in the study (Table 1) as controls. Patients were on average younger than the control group (62 ± 7.4 years for RHD vs. 72 ± 6.9 years for controls; *t*_(13.48)_ = −2.96, *p* = 0.01). The two groups did not differ in terms of formal education (10.5 ± 5.5 years for RHD vs. 11.42 ± 5.3 years for controls; *t*_(12.95)_ = −0.36, *p* = 0.732).

**Table 1:** Demographic (all participants) and clinical (RHD patients only) data.

Subject/group	Gender/age/education (years)	Handedness	Etiology	Lesional volume (cc)	Time from stroke (days)
1/RHD	F/63/5	R	I	172	221
2/RHD	M/60/8	R	H	684	1907
3/RHD	F/63/13	R	H	109	672
4/RHD	M/58/16	R	I	119	266
5/RHD	M/57/8	R	I	113	91
6/RHD	M/68/18	R	H	126	69
7/RHD	M/58/17	R	I	231	63
8/RHD	F/56/18	R	I	167	183
9/RHD	M/65/5	R	I	182	165
10/RHD	F/81/5	R	I	34	207
11/RHD	M/52/8	R	H	n.a.	72
12/RHD	F/63/5	R	I	292	192
1/Control	F/66/13	R			
2/Control	M/85/17	R			
3/Control	F/65/8	R			
4/Control	F/72/5	R			
5/Control	F/68/15	R			
6/Control	F/72/17	R			
7/Control	M/76/5	R			

*I, ischemic; H, hemorragic.*

Inclusion criteria for the clinical group were: the presence of a first-ever right-hemisphere stroke and severely impaired performance in detecting contralesional targets (accuracy below 25%) in face of a seemingly preserved performance in detecting ipsilesional ones (accuracy above 75%, see detailed operationalization later). Inclusion criterion for the control group was the absence of neurological disorders assessed with an extensive interview. Exclusion criteria for both groups were the presence of additional neurological/psychiatric disorders or substance abuse.

### Brain Lesions Reconstruction

**FIGURE 1:**
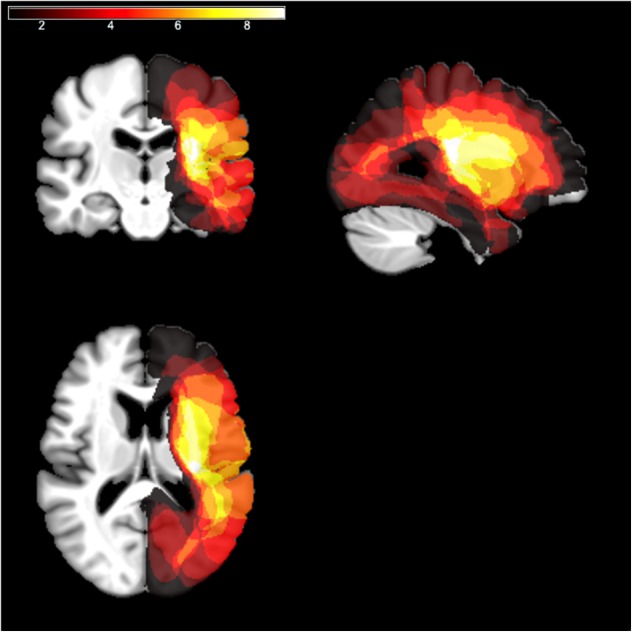
Lesion overlays. The lesion mapping for RHD patients, normalized to a template of aged healthy individuals according to the procedure described in Rorden et al. (2012), is shown as an overlay on a standard template using MRIcron (Rorden and Brett, 2000). The different colors code for the number of overlapping lesions from dark red (minimal/no overlap) to white (maximal overlap).

Individual scans (MRI or CT) were available for 11 patients out of 12. Brain lesions were automatically reconstructed with the software Lesion Identification with Neighborhood Data Analysis – LINDA (Pustina et al., 2016). Each reconstruction was independently checked by two experimenters and, if necessary, manually corrected using MRIcron (Rorden and Brett, 2000). Individual scans were reoriented using SPM (Friston et al., 2007) and then normalized to an age-appropriate template brain by means of the SPM Clinical Toolbox (Rorden et al., 2012) using enantiomorphic normalization (Nachev et al., 2008). Lesion overlays are depicted in Figure 1. The maximum overlap occurred for *n* = 9 patients in the right insula (X: 31, Y: −20, Z: 17).

### Neuropsychological Assessment

All patients underwent neuropsychological evaluation as per routine clinical practice (Table 1). The conventional part of the Behavioral Inattention Test (BIT) (Wilson et al., 1987) was administered for assessing visuo-spatial abilities. It includes six subtests: lines, letters, and stars cancellation, line bisection, figure copy, and spontaneous drawing. Each subtest was scored separately and contributed to form a global index of lateralized visuo-spatial performance. Six patients showed left neglect according to the BIT overall cut-off score (130). Scores in Table 2 are reported separately for right- and left-sided targets.

**Table 2:** Neuropsychological tests.

Subject/group	MMSE cut-off: 24	RAVEN cut-off: 18.96	BIT cut-off: <130	BIT-barrage	BIT-stars	BIT-letters
						
				L/R	L/R	L/R
						
				(max 18/18)	(max 27/27)	(max 20/20)
1/RHD	n.a	22,3	119^∗^	18/18	27/27	5/20
2/RHD	19^∗^	n.a	133	18/18	23/27	18/18
3/RHD	25,2	26,4	140	18/18	27/27	20/18
4/RHD	23,2^∗^	25,2	124^∗^	18/18	24/24	14/20
5/RHD	25	27,8	66^∗^	12/18	0/12	3/19
6/RHD	24,2	18,4^∗^	132	18/17	23/27	20/18
7/RHD	25,2	26,2	107^∗^	18/18	0/25	19/19
8/RHD	30	29,6	144	18/18	27/27	20/20
9/RHD	22,9^∗^	27,9	107^∗^	18/18	12/20	16/20
10/RHD	25,4	23	136	18/18	27/27	16/18
11/RHD	27	33,33	141	18/18	26/27	19/20
12/RHD	25,9	27	102^∗^	18/18	15/18	14/13

*MMSE [Mini Mental State Examination (Magni et al., 1996), and Raven’s progressive matrices (Carlesimo et al., 1996)]. Across all tasks, age and education corrected scores are reported. ^∗^Performance below cut-off. BIT [Behavioral Inattention Test (Wilson et al., 1987)]: scores at cancellation subtests are reported separately for left (L)/right (R) space. −: data not available. n.a.: unable to assess.*

### Preliminary Task for Study Inclusion

#### Stimuli and Procedure

**FIGURE 2:**
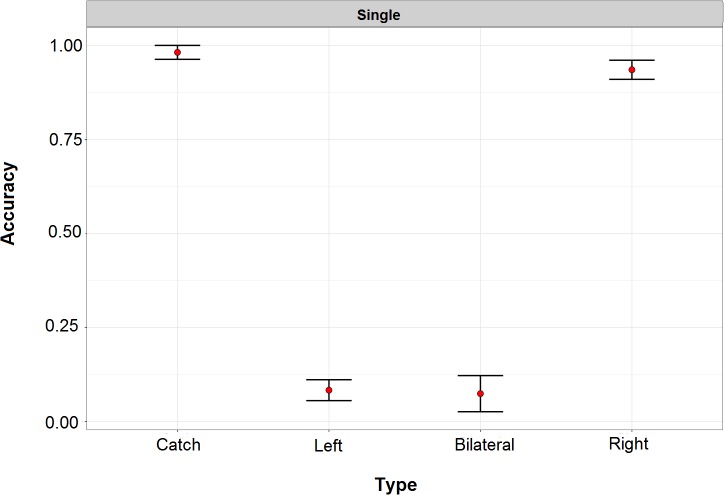
Performance of right hemisphere damaged patients in the preliminary task for study inclusion. In this task left and right refers to the two sides of the screen. Performance for left and double target was severely compromised. Across all patients, there was no response bias and detection of targets presented within the right side of space approached ceiling performance.

Patients were individually tested in a quiet room, sitting comfortably at a distance of about 60 cm from a 19-inch computer monitor. The task was adapted from Blini et al. (2016). Each trial started with a black screen (1000 ms), followed by a white fixation cross (about 1° wide) that was presented in the center of the screen for 800 ms. The lateralized visuospatial target was a white disk (diameter: 0.8°) presented against a black background for a duration of about 110 ms. The target could appear unilaterally, on the left or the right side of the display (distance from fixation: 17°), or bilaterally (both on the left- and on the right- side), simultaneously. To assess any potential response bias we included “Catch” trials, in which no target was actually displayed on the screen. The three target locations (left, right, bilateral) and the catch trials were random and equiprobable (i.e., 25% of each type). Simultaneously with the lateralized target(s), a visual shape (triangle, square, or circle) was shown at fixation, and an environmental sound (train whistle, doorbell, or hammer) was presented through binaural earphones. Once the 110 ms time window elapsed, a noisy screenshot was presented until the beginning of the following trial, as to minimize retinal after-image. Patients had to report the position of the target(s) (i.e., “no target,” “right,” “left,” or “both” sides). In total, 36 trials were presented. Selected patients correctly detected, at the group level, only 8% of left targets and 7% of bilateral ones. There was no response bias (accuracy to catch trials > 98%), and performance for right targets was highly accurate (94% of correct responses). Performance in this preliminary task is represented in Figure 2.

### Experimental Task

#### Stimuli and Procedure

**FIGURE 3:**
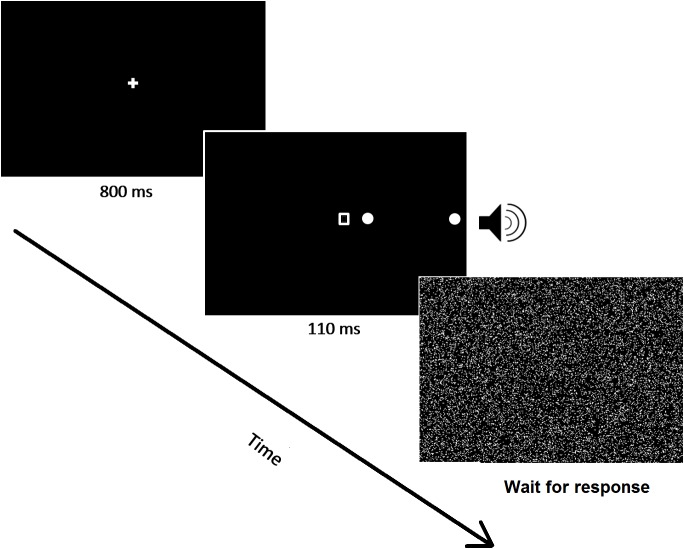
A schematic image of a representative trial (double target) is shown. All stimuli were presented within the ipsilesional visual field.

Patients omitting at least 75% of left, unilateral targets and less than 25% of ipsilesional targets in the preliminary task were included in the study and performed the experimental task. Task timing and stimuli were identical to those described above. Their position, instead, was different because the experimental task was specifically designed to test spatial awareness within the right side of space (see Figure 3). Lateralized targets were thus presented on the right of the fixation point either near right (3°), or far right (17°) (low vs. high eccentricity), or simultaneously in both locations (double target). Catch trials, in which no visual target was presented, were also administered to assess for any potential response bias. As in the screening task described above, a geometrical shape was always presented at fixation, coupled with the auditory presentation of an environmental sound (train whistle, doorbell, or hammer). There were three experimental conditions: one single-task condition, and two dual-task conditions (visual and auditory). Reporting target(s) position was the only request for the single task condition, whereas in the dual-visual or dual-auditory conditions patients also had to report, after having responded to the target, the central shape or the presented sound, respectively. The sensory stimulation was therefore kept identical across the three conditions, while the experimental manipulation was purely top–down, based on the nature and presence/absence of concurrent task demands.

Participants were allowed to rest after each trial, if necessary. The experimenter monitored eye movements and started each trial only when fixation was maintained. Trials affected by eye movements (<1%) were marked and discarded offline in the data analyses. The experiment was divided in 6 blocks, and each condition (single, auditory, or visual) was repeated twice (i.e., two blocks per condition). The single task condition was administered in the first and in the last block, in order to assess the potential effects of fatigue or sustained attention problems. The dual task conditions were performed in blocks 2 to 5 – with a fixed alternating order (i.e., visual-auditory-visual-auditory). A practice phase, consisting of 21 trials, was carried out before starting the experiment and allowed patients to familiarize with the task. Each experimental block comprised 36 trials. All possible combinations of shapes (3) and sounds (3) were presented within each block, balanced in frequency and with randomized order. Overall, the experiment consisted of 216 trials (3 load conditions × 4 types of target × 18 trials per cell) and could be completed in about 30 min by both patients and controls.

## Results

Analyses were performed using R version 3.5.1 (R Core Team, 2018). The following packages were used to implement data preprocessing and the pipeline for statistical analyses: dplyr v. 0.7.6 (Wickham et al., 2015); ggplot2 v. 3.0.0 (Wickham, 2016); afex v. 0.21-2 (Singmann et al., 2018); lme4 v. 1.1-17 (Bates et al., 2014, 2015b).

### Mixed Models on Accuracy

**FIGURE 4:**
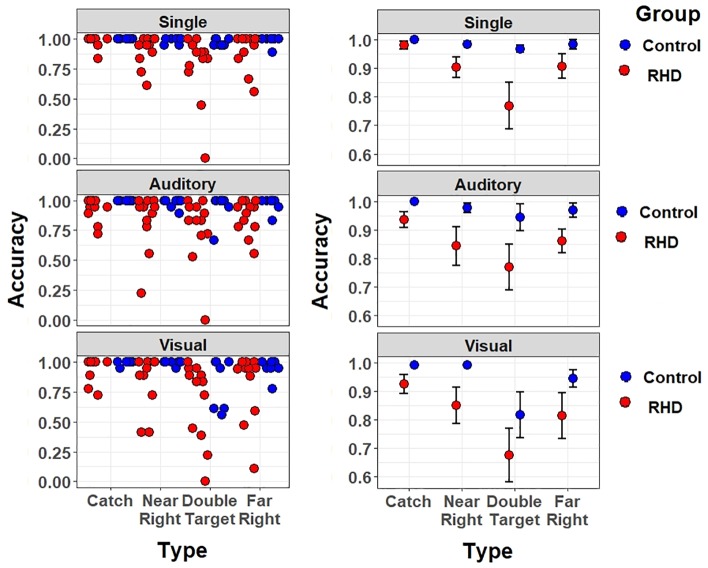
Accuracy effect for each target type and task (top-to-bottom: single task, auditory dual-task, and visual dual task), separately for right-hemisphere damaged patients (in red) and healthy controls (in blue). Left (right) panels show individual (mean ± SEM) performance.

Data have been first analyzed through *mixed-effects multiple regression models* (Baayen et al., 2008). A main advantage of mixed models is that they use single trial (rather than averaged) data; moreover, they do not assume independence amongst observations and the model fitting procedure takes into account the individual variability (random effects). This approach is particularly interesting for the analysis of clinical data because they are noisier than the data of healthy participants (for previous applications to stroke patients see, e.g., Zorzi et al., 2012; Blini et al., 2016). Models assessed detection accuracy as dependent variable through a logistic link-function, appropriate for binary variables. Prior to fixed-effect testing, the most appropriate and parsimonious (Bates et al., 2015a) matrix of random effects was chosen via an objective pipeline detailed at length in previous work (Blini et al., 2018). This pipeline for testing random effects suggested a hierarchical solution: subjects were specified as random intercepts, but nested in the respective Group, as this grouping accounted for significant variance in baseline performances. Furthermore, the random slope for stimulus Type was selected: this allows one to account, in the models, for the individual variability in performances across different configurations of stimuli. Note that the “Catch” trial Type had to be discarded from these analyses because characterized by a performance at near ceiling in both groups (see Figure 4), and thus yielded several convergence problems.

The testing of fixed effects found a significant main effect of Group [χ^2^(1) = 6.55, *p* = 0.01]. The average performance of patients was less accurate than that of controls (accuracy, excluding catch trials: 82.2 vs. 95.3%; odd ratio = 0.1, *SE* = 0.04). Furthermore, a significant main effect of Load was found [χ^2^(2) = 32.98, *p* < 0.001]; specifically, the Visual Dual-task impaired detection performance with respect to both the Single (odd ratio = 0.054, *SE* = 0.018, Wald *z* = 3.08, *p* = 0.006) and Auditory dual task (odd ratio = 0.039, *SE* = 0.016, Wald *z* = 2.35, *p* = 0.0496), with no differences between the latter two. There were no other significant effects or interactions (all *p*s > 0.085), showing that visual multitasking-induced performance decrements were comparable across Groups. Thus, when taking into account the number of errors and not their nature, the Visual dual-task condition was found to be equally challenging in both Groups.

However, the crucial test of this study concerns potential perceptual asymmetries in detecting targets in one or another spatial location. We therefore proceeded with a fine-grained analysis of Asymmetry Indices (AIs), which better inform about the presence of *lateralized* response biases (see below). Because controls performed at near ceiling, we focus on assessing lateralized biases in patients (mean accuracy of 82.2%).

### Lateralized Effects of Attentional Load on Spatial Monitoring

**FIGURE 5:**
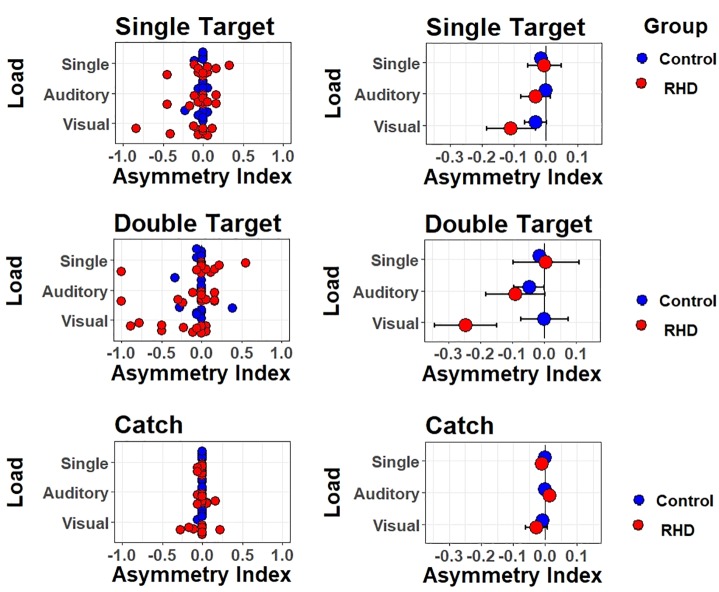
Lateralized effects on spatial monitoring performance. Asymmetry indexes are shown for each type of trial (one target, two targets, no target) and type of task/attentional load (single task, auditory dual task, visual dual task). Left (right) panels show individual (mean ± SEM) performance.

Asymmetry Indices summarize response asymmetries found when comparing the detection rate of ipsilesional less vs. more lateralized targets (see Figure 5). The AIs for double target and catch trials were (separately) computed by subtracting, for each individual, the proportion of “near right” [relative left] responses from the proportion of “far right” responses [relative right]. A negative AI indexes that “near right” responses prevailed among errors while positive AI reveals prevalence of “far right” responses. For unilateral trials, AIs were obtained by subtracting the proportion of omissions for far right targets from the proportion of omissions for near right targets. The unilateral AI is similar to the previous one, with negative values representing a leftward bias and positive values representing a rightward bias. AI values express here the asymmetry in terms of lateralized proportion of errors. That is, a value of −1 indicates that all (and only) the far right targets were missed, whereas a value of 0 indicates that an equal number of near right and far right targets were missed (or that no targets were missed). These three dependent variables were then submitted to a three-way ANOVA using Task (Single, Dual Visual, Dual Auditory) as independent variable.

For the patients group, results were as follows. For catch trials, the pattern of responses was not modulated by Task (*F*_(2,22)_ = 0.9, *p* = 0.42); the average AI (collapsed across Tasks) was not lateralized [*t*(11) = −0.57, *p* = 0.58], showing no evidence of a general response bias. For unilateral trials, results were similar, with no modulation by Task (*F*_(2,22)_ = 2.6, *p* = 0.097), and no lateralized bias on average [*t*(11) = −0.92, *p* = 0.38]. For double targets, however, Task induced a significant modulation (*F*_(2,22)_ = 6.11, *p <* 0.01). Follow-up *t*-tests showed that the AIs were significantly lateralized and negative (i.e., biased to the left) in the Visual dual-task [*t*(11) = −2.53, *p* = 0.028]. Paired *t*-tests further showed that AIs differed, and were more strongly left-lateralized, for both dual tasks with respect to the single task [single vs. Dual Visual *t*(11) = 2.9, *p* = 0.015; single vs. Dual Auditory *t*(11) = 2.24, *p* = 0.047]. The two dual tasks did not differ though, *t*(11) = 1.94, *p* = 0.08. This suggests that impairments emerged in the presence of a visual or auditory load and of double targets. Finally, we performed exploratory correlations between AI and both neuropsychological scores (i.e., BIT) and lesion volume, but no significant associations emerged.

### Fatigue and Sustained Attention

A specific analysis performed at the patients’ group level assessed whether fatigue, or deficient sustained attention, had an impact on performance accuracy. The single task was performed both at the beginning (i.e., first block of trials) and at the end (i.e., last block of trials) of the experiment. Therefore, a significant drop in accuracy between the first and the last block would suggest the presence of a confound due to fatigue.

**FIGURE 6:**
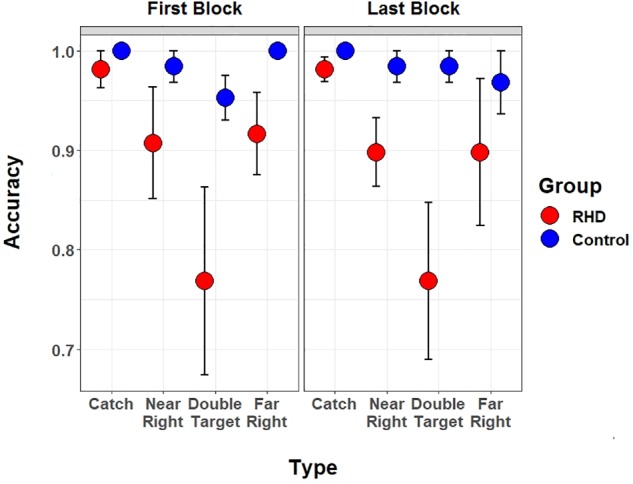
Blockwise Task performance. Performance of right hemisphere damaged patients (in red) and controls (in blue) is shown separately for the first and the last (i.e., sixth) block of the spatial monitoring task. In both blocks the single task version was performed (participants only had to report target position).

A 4 (Type: catch, near right, far right, or double target) by 2 (Session: first or last) mixed model, with the same analytic precautions described above, was therefore computed. Models included a random intercept for Subject and a random by-subject slope for Type. However, the analyses did not highlight effects of Session, as either a main effect nor in interaction with Type (all χ^2^ < 0.19, all *p*s > 0.69) (see Figure 6).

## Discussion

We investigated whether and how multitasking affects the conscious perception of ipsilaterally-presented single and double targets. The task was performed by chronic right-hemisphere stroke patients who were specifically selected because unable to perceive items in the contralesional side of space. Targets within the right, ipsilesional space could be presented either pericentrally (near right), more laterally (far right), or in both positions. We found that patients’ performance was particularly impaired for the most lateralized ipsilateral target position when the task required parallel processing of multiple stimuli, that is a double target and concurrent visual or auditory load.

This pattern of findings excludes the possibility that ipsilesional processing might become, after brain damage, hyperefficient due to attentional imbalance. Rather, it characterizes the performance for the ipsilesional space, or at least for the most lateralized part of the ipsilesional space, as impaired (see section “Introduction”). Strong support for this possibility comes, at individual level, from the pattern presented by Case 5. He was the only patient in the sample who presented a contralesional deficit so severe to result, in the STAR cancellation subtest, in omitting not only all targets in the left hemispace but also some on the right, ipsilesional, hemispace. In other words, this patient neglected a portion of space that extended (at least under demanding testing) to a visual angle corresponding to the position where the near-right ipsilesional targets were presented in the computer-based task. Despite this severe deficit in the cancellation task, his performance in the experimental task with double stimuli and visual load was characterized by systematic omission of the rightmost but not the leftmost target (AI: −0.77). The correct detection of targets in a spatial position that was neglected in the paper-and-pencil test occurred despite the brief duration of targets in the computer-based task.

An alternative explanation, which can only be partly ruled out here, claims that RHD patients (with neglect) may also present a severe bias favoring stimuli presented at fixation (Ptak et al., 2007). Distracters presented para-foveally severely disrupt saccade initiation irrespectively of saccade direction, leading to disproportionately increased latencies. According to Ptak et al. (2007), RHD patients with neglect not only fail to inhibit reflexive orienting toward ipsilesional items, but also exhibit a strong oculomotor bias favoring the fixated stimuli. According to this reasoning, one might claim that the impaired performance found for the rightmost targets was, at least in part, triggered by the presence of one central shape. While we cannot exclude this explanation, it seems worth pointing out that the central shape was already present under single task, when no asymmetry emerged. It rather seems that focusing attention on the central shape had to be considered a condition necessary but not sufficient for ipsilesional omissions to occur. In principle, the deficits might be ascribed to unspecific impairments. It should be also considered, however, that both alternative explanations are consistent with the presence of impaired mechanisms of visuo-spatial processing within the ipsilesional space.

Whether and how it is possible to isolate an advantage in ipsilesional processing without re-referencing performance to the contralesional side of space remains an open issue. Ipsilesional biases have been described as being predictive of long-term deficits, altogether with unspecific slowing (Viken et al., 2014). The clinical value of these findings perfectly summarizes the possibility that, also within a context of lateralized deficits, performance can be heavily affected by unspecific impairments. Support for the interaction between specific and unspecific factors comes from the study of spatial perseverations in drawing and cancellation tests. Despite having a clear spatial gradient, perseverations are more commonly due to a complex interaction between spatial and non-spatial components (Nys et al., 2010). Ronchi et al. (2009) showed that the degree of perseverations could be explained neither by neglect severity nor by executive functions deficits alone (also see Pia et al., 2009). One could therefore wonder whether, also in a purely perceptual domain, the same explanation holds. This would be only in part at odds with the idea that ipsilesional processing is related to neglect severity because non-spatial impairments are also directly related to neglect severity. Finally, it seems difficult to disentangle whether this putatively more effective performance is due to a sort of lack of inhibition, as it seems the case when ipsilesional stimuli are not task-relevant (Ptak et al., 2007). The functionality of the left attentional network might provide a compensatory effect after critical right-hemisphere lesions and be relevant for contralesional spatial processing (Blini et al., 2016).

Overall, results suggest that the approach we used in the present study is promising for detecting asymmetries in spatial monitoring caused by lateralized brain damage, as previously observed in chronic RHD patients (Bonato et al., 2010). The presence of omissions under load is all-but a curiosity. A wealth of studies (Ball and Owsley, 1993; Owsley et al., 1998; Ball et al., 2002) demonstrated that the “shrinkage” of visual field under visual load reliably predicts functional impairment. The amplitude of this impairment correlates with important everyday life outcomes such as for instance the risk of car crashes (Owsley et al., 1998).

Given the ubiquity of multitasking in everyday activities, and the practical impossibility to test performance within the contralesional hemispace in a number of patients (e.g., those with severe neglect and/or hemianopia), this approach can provide information that is clinically relevant (see Bonato et al., 2012).

The present study suggests that awareness disorders might not be only present in a contralesional to ipsilesional gradient. Instead, they might be present even for the most lateralized ipsilesional space portions, at least in the most demanding task conditions. This finding is particularly interesting because it contrasts two widely held aspects of ipsilesional processing. First, it is at odds with the evidence (collected in the absence of dual-tasking) suggesting that the most ipsilesional spatial positions are processed flawlessly even by the most severe neglect patients (Smania et al., 1998). Second, it seems incompatible with the possibility that ipsilesional items trigger an automatic orienting of attention toward them. The fact that the most demanding condition resulted in omissions fits with the hypothesis that the individual spatial pattern of awareness is determined by an interaction between a generalized lack of (non-spatial) resources and a more specific spatial processing deficit (Robertson et al., 1998; Bonato et al., 2010; Corbetta and Shulman, 2011). Once more, dual-tasking exacerbated a spatial deficit (ipsilesional, in this specific context) which was not detectable under single-task conditions and was present only for the most demanding conditions.

Whether the origin of these space-based consciousness disorders are unspecific deficits or whether it is, rather directly, linked to the severity of the spatial impairments, remains undetermined. It would be interesting to couple a modified version of the present task with rehabilitation trials for contralesional visual (Casco et al., 2018) or attentional deficits (Antonucci et al., 1995), to quantify the extent of subtle neglect deficits, to monitor changes over time, and also to assess the effectiveness of rehabilitation (Azouvi, 2017; Chen et al., 2017).

The present study is a first attempt to explore the effects of multitasking in ipsilesional hemispace and has several limitations. First, our small sample size is more prone to a descriptive/qualitative approach. Second, we lack information as to whether homonymous hemianopia was present in patients (beyond the simple clinical testing with single and double simultaneous stimulation). There is no doubt that it would have been interesting to know whether the individual ipsilesional impairment was associated with a contralesional visual field deficit. For the sake of completeness, however, it should be mentioned that our patients were all functionally blind for the contralesional side of space and that a visual field assessment is often not sufficient to determine whether a patient suffers from hemianopia or “only” from severe neglect (Walker et al., 1991; Müller-Oehring et al., 2003). The third, and most important weakness is the absence of a control group of left-hemisphere damaged patients. Without such a reference it seems difficult to understand whether the deficits we preliminarily highlighted in the present study are specific or unspecific consequences of RHD. For future studies, it would be also interesting to more extensively map several eccentricities rather than only two.

In short, by using an adapted version of a multitasking approach we explored ipsilesional spatial awareness after right-hemisphere stroke in patients who could not detect the presence of briefly-presented, contralesional items. This promising approach allowed us to discard the possibility that stroke might preserve ipsilesional performance or make it hyper-efficient. Rather, systematic errors were found in the patients group in the case of two targets being simultaneously presented. When concurrent information had to be processed at fixation, several of the more lateralized items appearing within the putatively unaffected visual hemispace went unreported in patients but not in healthy controls.

## Ethics Statement

The study and all procedures were approved by the Ethics Committee for Clinical Research (CESC) of the Venice region (Reference No. 2014.09) and were carried out in accordance with the Declaration of Helsinki. All participants gave written informed consent to take part into the study.

## Author Contributions

MB, ZR, EB, MP, and MZ conceived the study. ZR, ED, and LP collected the data. FM and LP supervised patient recruitment. ZR and EB performed the analyses. MB drafted the manuscript, with contributions from ZR, EB, and MZ. All authors reviewed the manuscript.

## Conflict of Interest Statement

The authors declare that the research was conducted in the absence of any commercial or financial relationships that could be construed as a potential conflict of interest. The handling Editor declared a shared affiliation, though no other collaboration, with one of the authors MP at the time of review.
