# Supplementary material for: Ipsilesional Impairments of Visual Awareness After Right-Hemispheric Stroke
Source: Front Psychol. 2019 Apr 9;10:697. doi: 10.3389/fpsyg.2019.00697 (PMC6465520; doi:10.3389/fpsyg.2019.00697)
Supplement: Supplementary file 1 [file Data_Sheet_1.zip › Supplementary_Bonato_et_al_Frontiers.html]

Supplementary Materials Frontiers


# Supplementary Materials Frontiers

#### *Bonato, Romeo, et al.*

#### *06 marzo 2019*

This script includes the raw data and R scripts for reproducing the analyses in the paper.

Please refer to the manuscript for information about the task.

You can import this file directly in R or use the function `load(file.choose()`.

# Pre processing

We will need the following packages:

```
packages = c("ggplot2",
             "ez",
             "plyr",
             "lme4",
             "afex",
             "magrittr",
             "dplyr",
             "gridExtra",
             "emmeans")
             ##load them
             lapply(packages, require, character.only = T)
```

```
## Warning: package 'ggplot2' was built under R version 3.5.2
```

```
## Warning: package 'lme4' was built under R version 3.5.2
```

```
## Warning: package 'afex' was built under R version 3.5.2
```

```
## Warning: package 'dplyr' was built under R version 3.5.2
```

```
## Warning: package 'emmeans' was built under R version 3.5.2
```

```
## [[1]]
## [1] TRUE
## 
## [[2]]
## [1] TRUE
## 
## [[3]]
## [1] TRUE
## 
## [[4]]
## [1] TRUE
## 
## [[5]]
## [1] TRUE
## 
## [[6]]
## [1] TRUE
## 
## [[7]]
## [1] TRUE
## 
## [[8]]
## [1] TRUE
## 
## [[9]]
## [1] TRUE
```

In this part we will translate italian names into english, but also declare several variables as factors or numeric when needed.

```
#create, define, or rename variables
## Group as factor, then reorder
data$Group = factor(data$Group, levels = targetGroups[, 1])

##rename variables
###define custom function for this task
###change Italian names into English-friendly ones
translate.names = function(old.name, new.name, DF = data) {
names(DF)[names(DF) == old.name] = new.name
assign("data", DF, envir = .GlobalEnv)
}

###Subjects' response
translate.names("RispostaPosizione.RESP", "Response")
###Correct response
translate.names("RispostaPosizione.CRESP", "CorrectResponse")
###Side/Type stimuli
translate.names("LatoComparsa", "Type")
####rename levels
if (sum(levels(data$Type) == c("ambo", "catch", "dx", "sx")) == 4) {
levels(data$Type) = c("Bilateral", "Catch", "Right", "Left")
} else {
warning("Unexpected order of levels, please check data$Type")
}
####order levels
data$Type = factor(data$Type, c("Catch", "Left", "Bilateral", "Right"))
###Accuracy first task
####check
sum(!ifelse(data$Response == data$CorrectResponse, 1, 0) ==
data$RispostaPosizione.ACC) == 0
```

```
## [1] TRUE
```

```
translate.names("RispostaPosizione.ACC", "AccuracyPosition")

##create variables
###create LOAD variable... Could not find a more elegant way...
data$Load = NA
data$Load[data$ExperimentName == "Dual Uditivo" |
data$ExperimentName == "Dual Uditivo 2"] <- "Auditory"
data$Load[data$ExperimentName == "Dual Visivo" |
data$ExperimentName == "Dual Visivo 2"] <- "Visual"
data$Load[data$ExperimentName == "Singolo" |
data$ExperimentName == "Singolo 2"] <- "Single"
data$Load = factor(data$Load)
data$Load = relevel(data$Load, "Single")

###Accuracy to secondary task (auditory/visual only)
data$AccuracyST = ifelse(data$Load == "Auditory",
data$RispostaSuono.ACC,
data$RispostaForma.ACC)

data$AccuracyST = as.numeric(ifelse(
data$Load == "Auditory",
as.character(data$RispostaSuono.ACC),
as.character(data$RispostaForma.ACC)
))
###Session
data$Session = data$ExperimentName
data$Session = revalue(
data$Session,
c(
"Singolo" = "1",
"Singolo 2" = "6",
"Dual Uditivo" = "3",
"Dual Uditivo 2" = "5",
"Dual Visivo" = "2",
"Dual Visivo 2" = "4"
)
)

###Eye movements FirstTask
data$EyeMovementsFT = ifelse(data$Response == "p", 1, 0)
###Progressive ID number
data$ID = factor(as.factor(data$Subject))
levels(data$ID) = seq(1, to = length(levels(as.factor(data$ID))))
```

We quantify and exclude eye movements:

```
#quantify and then omit ocular movements
##factors used to quantify ocular movements by (e.g. by Subject, by Subject and Side...)
by = c("Subject", "Group")
###summarise
EM.dataframe = ddply(data, by, summarise, ProportionEyeMovements = mean(EyeMovementsFT))
###thus
mean(EM.dataframe$ProportionEyeMovements)
```

```
## [1] 0.002436647
```

```
tapply(EM.dataframe$ProportionEyeMovements,
EM.dataframe$Group,
mean)
```

```
##         RHD     Control 
## 0.003858025 0.000000000
```

```
##omit responses with deviant eye movements
data = data[data$EyeMovementsFT == 0, ]
```

# Summary indices

We summarize accuracy in various dataframes:

```
#accuracies - position
##for analyses
ACC.subjects = ddply(
data,
c("ID", "Subject", "Group", "Load", "Type"),
summarise,
AccuracyPosition = mean(AccuracyPosition)
)
ACC.subjects$Group = factor(ACC.subjects$Group, levels = targetGroups[, 1])
##for plotting
ACC = ddply(
ACC.subjects,
c("Group", "Load", "Type"),
summarise,
Accuracy = mean(AccuracyPosition),
N = length(ID)
)
ACC$SD = ddply(ACC.subjects,
c("Group", "Load", "Type"),
summarise,
SD = sd(AccuracyPosition))$SD
ACC$SEM = ACC$SD / sqrt(ACC$N)
ACC$Group = factor(ACC$Group, levels = targetGroups[, 1])

ACC.subjects2 = ACC.subjects[!ACC.subjects$Type == "Catch", ]
ACC.subjects3 = ACC.subjects[!ACC.subjects$Type == "Bilateral", ]
```

Accuracy for each session (fatigue effect):

```
#accuracies - session
##for analyses
ACC.subjects.session = ddply(
data[data$Session == "1" | data$Session == "6", ],
c("ID", "Group", "Type", "Session"),
summarise,
AccuracyPosition = mean(AccuracyPosition)
)
##for plotting
ACC.session = ddply(
ACC.subjects.session,
c("Group", "Type", "Session"),
summarise,
Accuracy = mean(AccuracyPosition),
N = length(ID)
)
ACC.session$SD = ddply(
ACC.subjects.session,
c("Group", "Type", "Session"),
summarise,
SD = sd(AccuracyPosition)
)$SD
ACC.session$SEM = ACC.session$SD / sqrt(ACC.session$N)
ACC.session$Group = factor(ACC.session$Group, levels = targetGroups[, 1])
```

Asymmetry indices:

```
#define a function to compute asymmetries
##this is for bilateral (target==2) or catch trials (target== 0)
lat.index <- function(DF, target) {
#subset of responses of the correct type
DF <- DF[DF$CorrectResponse == target, ]
R = 0
L = 0
R = sum(DF$Response == 3) / nrow(DF) #proportion of responses right
L = sum(DF$Response == 1) / nrow(DF) #proportion of responses left
L.I = (R - L)
ifelse(is.nan(L.I), L.I <- 0, L.I <- L.I)
return(as.numeric(as.character(L.I)))
}

##omission asymmetry to unilateral targets
omission.index <- function(DF) {
#subset of responses of the correct type
DF <- DF[DF$CorrectResponse == 1 | DF$CorrectResponse == 3, ]
#proportion of left omissions
DF1 <- DF[DF$CorrectResponse == 1, ]
L = sum(DF1$Response == 0) / nrow(DF1)
#proportion of right omissions
DF2 <- DF[DF$CorrectResponse == 3, ]
R = sum(DF2$Response == 0) / nrow(DF2)

L.I = (L - R)
ifelse(is.nan(L.I), L.I <- 0, L.I <- L.I)
return(as.numeric(as.character(L.I)))
}


## the following function requires a dataframe, an ID number to compute asymmetries for
## and grouping variables if any (e.g. Load), but you might want to add something else
## it returns a dataframe with asymmetries for Unilateral (Omission index), bilateral, catch trials
## the assumption is that variables in your dataframe are named as above
asymmetry.indices = function(ID, by = "Load", DF = data) {
#first, a subset of the given DF is taken
DF = DF[DF$ID == ID, ]

#then a vector for all the possible interactions given by "by" is given
interaction = interaction(DF[, by])
levels(interaction) = 1:length(levels(interaction))

#empty dataframe to store data
results = {
}
for (i in 1:length(levels(interaction))) {
#further subset
X = DF[interaction == i, ]

A = cbind(
ID = ID,
OriginalID = as.character(DF$Subject[1]),
Group = as.character(DF$Group[1]),
Case = as.character(levels(interaction(DF[, by]))[i]),
if ("Load" %in% by)
(Load = as.character(X$Load[1])),
Unilateral = omission.index(X),
Bilateral = lat.index(X, 2),
Catch = lat.index(X, 0)
)

results = rbind(results, A)
}

results = as.data.frame(results)
rownames(results) = NULL
to.numeric = c("ID", "Unilateral", "Bilateral", "Catch")
for (i in 1:length(to.numeric))
(
results[, to.numeric[i]] = as.numeric(as.character(results[, to.numeric[i]])))
if("Load" %in% by) (colnames(results)[colnames(results)=="V5"]= "Load")
return(results)
}

## now you can test each patient or participant like this:
asymmetry.indices(5)
```

```
##   ID OriginalID   Group     Case     Load Unilateral Bilateral Catch
## 1  5         73 Control   Single   Single          0         0     0
## 2  5         73 Control Auditory Auditory          0         0     0
## 3  5         73 Control   Visual   Visual          0         0     0
```

```
#compute asymmetries for each participant
Asymmetry = {
}
for (i in levels(data$ID)) {
Asymmetry = rbind(Asymmetry,
asymmetry.indices(i))
}
Asymmetry
```

```
##    ID OriginalID   Group     Case     Load  Unilateral   Bilateral
## 1   1          2     RHD   Single   Single -0.11111111  0.00000000
## 2   1          2     RHD Auditory Auditory -0.11111111 -0.11111111
## 3   1          2     RHD   Visual   Visual -0.05555556 -0.22222222
## 4   2         11     RHD   Single   Single  0.00000000  0.00000000
## 5   2         11     RHD Auditory Auditory  0.00000000  0.00000000
## 6   2         11     RHD   Visual   Visual  0.00000000  0.05555556
## 7   3         53     RHD   Single   Single  0.00000000  0.11111111
## 8   3         53     RHD Auditory Auditory  0.00000000  0.16666667
## 9   3         53     RHD   Visual   Visual  0.00000000  0.05555556
## 10  4         56     RHD   Single   Single  0.00000000  0.00000000
## 11  4         56     RHD Auditory Auditory  0.00000000  0.00000000
## 12  4         56     RHD   Visual   Visual  0.00000000  0.00000000
## 13  5         73 Control   Single   Single  0.00000000  0.00000000
## 14  5         73 Control Auditory Auditory  0.00000000  0.00000000
## 15  5         73 Control   Visual   Visual  0.00000000  0.00000000
## 16  6         74 Control   Single   Single  0.00000000  0.00000000
## 17  6         74 Control Auditory Auditory  0.05555556  0.00000000
## 18  6         74 Control   Visual   Visual  0.00000000  0.00000000
## 19  7         88 Control   Single   Single  0.00000000  0.00000000
## 20  7         88 Control Auditory Auditory  0.00000000  0.00000000
## 21  7         88 Control   Visual   Visual  0.05555556  0.00000000
## 22  8         89 Control   Single   Single -0.11111111 -0.05555556
## 23  8         89 Control Auditory Auditory  0.00000000 -0.33333333
## 24  8         89 Control   Visual   Visual -0.22222222 -0.27777778
## 25  9         96 Control   Single   Single  0.00000000  0.00000000
## 26  9         96 Control Auditory Auditory  0.00000000  0.00000000
## 27  9         96 Control   Visual   Visual  0.00000000  0.38888889
## 28 10         97 Control   Single   Single  0.00000000  0.00000000
## 29 10         97 Control Auditory Auditory -0.05555556  0.00000000
## 30 10         97 Control   Visual   Visual -0.05555556 -0.05555556
## 31 11        100 Control   Single   Single  0.00000000 -0.05555556
## 32 11        100 Control Auditory Auditory  0.00000000  0.00000000
## 33 11        100 Control   Visual   Visual  0.00000000 -0.05555556
## 34 12        105     RHD   Single   Single  0.05555556  0.05555556
## 35 12        105     RHD Auditory Auditory  0.16666667  0.16666667
## 36 12        105     RHD   Visual   Visual -0.11764706 -0.77777778
## 37 13        118     RHD   Single   Single  0.33333333  0.55555556
## 38 13        118     RHD Auditory Auditory  0.16666667  0.16666667
## 39 13        118     RHD   Visual   Visual -0.05555556 -0.11111111
## 40 14        120     RHD   Single   Single -0.05555556  0.16666667
## 41 14        120     RHD Auditory Auditory  0.00000000  0.00000000
## 42 14        120     RHD   Visual   Visual  0.05555556  0.00000000
## 43 15        121     RHD   Single   Single -0.44444444 -1.00000000
## 44 15        121     RHD Auditory Auditory -0.44444444 -1.00000000
## 45 15        121     RHD   Visual   Visual -0.83333333 -0.88888889
## 46 16        122     RHD   Single   Single  0.16666667  0.22222222
## 47 16        122     RHD Auditory Auditory -0.05555556  0.05555556
## 48 16        122     RHD   Visual   Visual  0.11111111  0.00000000
## 49 17        139     RHD   Single   Single  0.05555556  0.00000000
## 50 17        139     RHD Auditory Auditory -0.16666667 -0.23529412
## 51 17        139     RHD   Visual   Visual -0.41176471 -0.50000000
## 52 18        140     RHD   Single   Single -0.05555556  0.00000000
## 53 18        140     RHD Auditory Auditory  0.00000000  0.00000000
## 54 18        140     RHD   Visual   Visual  0.00000000 -0.05555556
## 55 19        145     RHD   Single   Single  0.00000000 -0.05555556
## 56 19        145     RHD Auditory Auditory  0.05555556 -0.29411765
## 57 19        145     RHD   Visual   Visual  0.00000000 -0.50000000
##          Catch
## 1   0.00000000
## 2  -0.05555556
## 3  -0.11111111
## 4   0.00000000
## 5   0.00000000
## 6   0.00000000
## 7   0.00000000
## 8   0.05555556
## 9   0.00000000
## 10  0.00000000
## 11  0.05555556
## 12  0.22222222
## 13  0.00000000
## 14  0.00000000
## 15  0.00000000
## 16  0.00000000
## 17  0.00000000
## 18 -0.05555556
## 19  0.00000000
## 20  0.00000000
## 21  0.00000000
## 22  0.00000000
## 23  0.00000000
## 24  0.00000000
## 25  0.00000000
## 26  0.00000000
## 27  0.00000000
## 28  0.00000000
## 29  0.00000000
## 30  0.00000000
## 31  0.00000000
## 32  0.00000000
## 33  0.00000000
## 34  0.00000000
## 35  0.00000000
## 36  0.00000000
## 37  0.00000000
## 38  0.00000000
## 39  0.00000000
## 40  0.00000000
## 41  0.00000000
## 42  0.00000000
## 43  0.00000000
## 44  0.00000000
## 45  0.00000000
## 46  0.00000000
## 47  0.00000000
## 48  0.00000000
## 49  0.00000000
## 50 -0.05555556
## 51  0.00000000
## 52 -0.05555556
## 53  0.00000000
## 54 -0.27777778
## 55 -0.05555556
## 56  0.16666667
## 57 -0.16666667
```

```
#reorder
Asymmetry$Load = factor(Asymmetry$Load, rev(c("Single", "Auditory", "Visual")))
Asymmetry$Group = factor(Asymmetry$Group, levels = targetGroups[, 1])

#summarise for plotting
Asymmetry.Means = ddply(
Asymmetry,
c("Group", "Load"),
summarise,
Unilateral = mean(Unilateral),
Bilateral = mean(Bilateral),
Catch = mean(Catch),
N = length(ID)
)
#standard deviations
Asymmetry.Means$SD.Unilateral = 0
Asymmetry.Means$SD.Bilateral = 0
Asymmetry.Means$SD.Catch = 0

Asymmetry.Means[, seq(ncol(Asymmetry.Means) - 2, ncol(Asymmetry.Means))] =
ddply(
Asymmetry,
c("Group", "Load"),
summarise,
SD.Unilateral = sd(Unilateral),
SD.Bilateral = sd(Bilateral),
SD.Catch = sd(Catch)
)[, 3:5]
#sems
Asymmetry.Means$SEM.Unilateral = Asymmetry.Means$SD.Unilateral / sqrt(Asymmetry.Means$N)
Asymmetry.Means$SEM.Bilateral = Asymmetry.Means$SD.Bilateral / sqrt(Asymmetry.Means$N)
Asymmetry.Means$SEM.Catch = Asymmetry.Means$SD.Catch / sqrt(Asymmetry.Means$N)

#reorder
Asymmetry.Means$Load = factor(Asymmetry.Means$Load, rev(c("Single", "Auditory", "Visual")))
```

# Plots

These will reproduce the figures in the paper.

Accuracy:

```
#ggplot theme, common to all plots
#color-blind friendly palette
cbPalette= c("red", "blue", "#56B4E9", "#009E73", "#F0E442", "#0072B2", "#D55E00", "#CC79A7")
commonTheme= list(theme_bw(),
                  theme(text= element_text(size=20, face="bold")),
                  scale_fill_manual(values= cbPalette))

#accuracy - all subjects
levels(ACC.subjects$Type)= c("Catch", "Near\nRight", "Double\nTarget", "Far\nRight")

pd= position_jitterdodge(dodge.width= 1, jitter.width= 0.8)

p1= ggplot(ACC.subjects, aes(x= Type, y= AccuracyPosition, group= Group, colour= Group, fill= Group)) + 
  commonTheme + facet_wrap("Load", nrow = 3) + ylab("Accuracy") +
  geom_point(position= pd, size= 4, shape= 21, colour= "black")

#accuracy - mean
levels(ACC$Type)= c("Catch", "Near\nRight", "Double\nTarget", "Far\nRight")

pd= position_dodge(0.4)

p2= ggplot(ACC, aes(x= Type, y= Accuracy, group= Group, fill= Group)) + 
  commonTheme +  
  geom_errorbar(position= pd, aes(ymin= Accuracy-SEM, ymax= Accuracy+SEM), 
                width= .3, colour= "black", size= 1) +
  facet_wrap("Load", nrow = 3) +
  geom_point(position= pd, size= 4, shape= 21, colour= "black")


#extract legend
#https://github.com/hadley/ggplot2/wiki/Share-a-legend-between-two-ggplot2-graphs
g_legend<-function(a.gplot){
  tmp <- ggplot_gtable(ggplot_build(a.gplot))
  leg <- which(sapply(tmp$grobs, function(x) x$name) == "guide-box")
  legend <- tmp$grobs[[leg]]
  return(legend)}

mylegend<-g_legend(p1)

grid.arrange(p1 + theme(legend.position="none"),
                   p2 + theme(legend.position="none"),
                   mylegend, layout_matrix= rbind(c(1, 1, 2, 2, 3)))
```

Asymmetry Indices.

```
ggplot.me.subjects= function(dv, DF= Asymmetry){
  
  p= ggplot(DF, aes(x= Load, y= get(dv), group= Group, colour= Group, fill= Group)) + 
    commonTheme +  ylab("Asymmetry Index") + ylim(c(-1, 1)) +
    geom_segment(y= 0, yend= 0, x= 0.5, xend= 3.5, colour= "black") +
    geom_point(position= pd, size= 4, shape= 21, colour= "black") + coord_flip() 
  
  if (dv== "Bilateral") (p= p + ggtitle("Double Target")) else {
    if (dv== "Unilateral") (p= p + ggtitle("Single Target")) else (p= p + ggtitle(dv))}
  
  return(p)   
}
ggplot.me= function(dv, DF= Asymmetry.Means){
  
  eb= paste0("SEM.", dv)
  
  p= ggplot(DF, aes(x= Load, y= get(dv), group= Group, fill= Group)) + 
    commonTheme +  ylab("Asymmetry Index") + ylim(c(-0.35, 0.15)) +
    geom_segment(y= 0, yend= 0, x= 0.5, xend= 3.5, colour= "black") +
    geom_errorbar(position= pd, aes(ymin= get(dv) - get(eb), 
                                    ymax= get(dv) + get(eb)), 
                  width= .3, colour= "black", size= 1) +
    geom_point(position= pd, size= 6, shape= 21, colour= "black") + coord_flip() 
  
  if (dv== "Bilateral") (p= p + ggtitle("Double Target")) else {
    if (dv== "Unilateral") (p= p + ggtitle("Single Target")) else (p= p + ggtitle(dv))}
  
  return(p)   
  
}

pd= position_dodge(0.4)
grid.arrange(ggplot.me("Unilateral"),
             ggplot.me("Bilateral"),
             ggplot.me("Catch"), 
             ncol=1, nrow =3)
```

```
#merge plots
pd= position_jitterdodge(dodge.width= 1, jitter.width= 0.8)
grid.arrange(ggplot.me.subjects("Unilateral"),
             ggplot.me.subjects("Bilateral"),
             ggplot.me.subjects("Catch"), 
             ncol=1, nrow =3)
```

```
#first solution
pd= position_jitterdodge(dodge.width= 1, jitter.width= 0.8)

p1= ggplot.me.subjects("Unilateral")
my_legend= g_legend(p1)
p1= p1 + theme(legend.position="none")

p2= ggplot.me.subjects("Bilateral") + theme(legend.position="none")

p3= ggplot.me.subjects("Catch") + theme(legend.position="none")

pd= position_dodge(0.4)

p4= ggplot.me("Unilateral") + theme(legend.position="none")

p5= ggplot.me("Bilateral") + theme(legend.position="none")

p6= ggplot.me("Catch") + theme(legend.position="none")

grid.arrange(p1, p2, p3, p4, p5, p6, my_legend,
             layout_matrix= cbind(c(1, 1, 2, 2, 3, 3),
                                  c(1, 1, 2, 2, 3, 3),
                                  c(4, 4, 5, 5, 6, 6),
                                  c(4, 4, 5, 5, 6, 6),
                                  c(NULL, NULL, 7, 7, NULL, NULL)))
```

Fatigue (thus, first vs last session):

```
levels(ACC.session$Type)= c("Catch", "Near\nRight", "Double\nTarget", "Far\nRight")


pd= position_dodge(0.8)
session_names= c("1" = "First Session", "6" = "Last Session")
ggplot(ACC.session, aes(x= Type, y= Accuracy, group= Group, fill= Group)) + 
  commonTheme +  
  facet_wrap(c("Session"), labeller= labeller(Session= session_names)) +
  geom_errorbar(position= pd, aes(ymin= Accuracy-SEM, ymax= Accuracy+SEM), 
                width= .3, colour= "black", size= 1) +
  geom_point(position= pd, size= 8, shape= 21, colour= "black") +
  ggtitle("Effect of Fatigue")
```

# Analyses

## Mixed models over accuracy.

As can be appreciated in the figures before, the catch trials were at ceiling for most of the subjects. We exclude them.

```
data_mm= data[!data$Type== "Catch",]
data_mm$Type= factor(data_mm$Type)
```

We start with selecting the most appropriate matrix of random effects.

```
mod0= glmer(AccuracyPosition ~ 0 + (1|Subject), 
            family = "binomial", data= data_mm)
mod0a= glmer(AccuracyPosition ~ 0 + (1|Group/Subject), 
            family = "binomial", data= data_mm)

anova(mod0, mod0a) #yes
```

```
## Data: data_mm
## Models:
## mod0: AccuracyPosition ~ 0 + (1 | Subject)
## mod0a: AccuracyPosition ~ 0 + (1 | Group/Subject)
##       Df    AIC    BIC  logLik deviance  Chisq Chi Df Pr(>Chisq)    
## mod0   1 2098.3 2104.3 -1048.1   2096.3                             
## mod0a  2 2071.4 2083.4 -1033.7   2067.4 28.892      1  7.651e-08 ***
## ---
## Signif. codes:  0 '***' 0.001 '**' 0.01 '*' 0.05 '.' 0.1 ' ' 1
```

```
mod0b= glmer(AccuracyPosition ~ 0 + (1+Type|Group/Subject), 
            family = "binomial", data= data_mm)

anova(mod0a, mod0b) #yes
```

```
## Data: data_mm
## Models:
## mod0a: AccuracyPosition ~ 0 + (1 | Group/Subject)
## mod0b: AccuracyPosition ~ 0 + (1 + Type | Group/Subject)
##       Df    AIC    BIC   logLik deviance  Chisq Chi Df Pr(>Chisq)    
## mod0a  2 2071.4 2083.4 -1033.69   2067.4                             
## mod0b 12 1797.8 1870.2  -886.91   1773.8 293.56     10  < 2.2e-16 ***
## ---
## Signif. codes:  0 '***' 0.001 '**' 0.01 '*' 0.05 '.' 0.1 ' ' 1
```

```
mod0c= glmer(AccuracyPosition ~ 0 + (1+Type+Load|Group/Subject), 
            family = "binomial", data= data_mm)

#convergence problems
```

Now we can switch to fixed effects. The all\_fit parameter takes long but checkes multiple optimizers and gets rid of possible convergence problems.

```
af19= mixed(AccuracyPosition ~ Group*Type*Load + (1+Type|Group/Subject), 
            family = "binomial", data= data_mm, all_fit = T, 
            type = 2, method = "LRT")
```

```
## Fitting 10 (g)lmer() models:
## [..........]
```

```
af19$anova_table
```

```
## Mixed Model Anova Table (Type 2 tests, LRT-method)
## 
## Model: AccuracyPosition ~ Group * Type * Load + (1 + Type | Group/Subject)
## Data: data_mm
## Df full model(s): 18
## Df full model(s): 18
## Df full model(s): 18
## Df full model(s): 26
## Df full model(s): 26
## Df full model(s): 26
## Df full model(s): 30
##                 Df   Chisq Chi Df Pr(>Chisq)    
## Group           17  6.5547      1    0.01046 *  
## Type            16  4.2124      2    0.12170    
## Load            16 32.9812      2   6.89e-08 ***
## Group:Type      24  0.0685      2    0.96634    
## Group:Load      24  3.3661      2    0.18580    
## Type:Load       22  8.1974      4    0.08461 .  
## Group:Type:Load 26  4.8535      4    0.30266    
## ---
## Signif. codes:  0 '***' 0.001 '**' 0.01 '*' 0.05 '.' 0.1 ' ' 1
```

Mean accuracy is:

```
data %>% 
  filter(! Type== "Catch") %>% 
  group_by(Group) %>% 
  summarise(M= mean(AccuracyPosition))
```

```
## # A tibble: 2 x 2
##   Group       M
##   <fct>   <dbl>
## 1 RHD     0.822
## 2 Control 0.953
```

And post-hocs:

```
emmeans(af19, pairwise ~ Group, transform= "response", data= af19$data)
```

```
## $emmeans
##  Group    prob     SE  df asymp.LCL asymp.UCL
##  RHD     0.876 0.0384 Inf     0.801     0.952
##  Control 0.978 0.0124 Inf     0.954     1.002
## 
## Results are averaged over the levels of: Type, Load 
## Confidence level used: 0.95 
## 
## $contrasts
##  contrast      estimate     SE  df z.ratio p.value
##  RHD - Control   -0.102 0.0403 Inf -2.520  0.0117 
## 
## Results are averaged over the levels of: Type, Load
```

```
emmeans(af19, pairwise ~ Load, transform= "response", data= af19$data)
```

```
## $emmeans
##  Load      prob     SE  df asymp.LCL asymp.UCL
##  Single   0.950 0.0167 Inf     0.918     0.983
##  Auditory 0.935 0.0185 Inf     0.899     0.971
##  Visual   0.896 0.0288 Inf     0.840     0.953
## 
## Results are averaged over the levels of: Group, Type 
## Confidence level used: 0.95 
## 
## $contrasts
##  contrast          estimate      SE  df z.ratio p.value
##  Single - Auditory   0.0155 0.00965 Inf 1.607   0.2426 
##  Single - Visual     0.0541 0.01755 Inf 3.083   0.0058 
##  Auditory - Visual   0.0386 0.01645 Inf 2.347   0.0496 
## 
## Results are averaged over the levels of: Group, Type 
## P value adjustment: tukey method for comparing a family of 3 estimates
```

## ANOVA on Asymmetry Indices

Function for ANOVA and multiple t-tests:

```
#let's define all the functions!

##multiple t vs 0, AI collapsed across load
manyttestv0collapsed = function(DF = Asymmetry) {
ddply(
Asymmetry,
c("Group", "OriginalID"),
summarise,
Unilateral = mean(Unilateral),
Bilateral = mean(Bilateral),
Catch = mean(Catch)
) %>%
ddply(
c("Group"),
summarise,
df.unilateral = t.test(Unilateral)$parameter,
t.unilateral = t.test(Unilateral)$statistic,
p.unilateral = t.test(Unilateral)$p.value,
df.bilateral = t.test(Bilateral)$parameter,
t.bilateral = t.test(Bilateral)$statistic,
p.bilateral = t.test(Bilateral)$p.value,
df.catch = t.test(Catch)$parameter,
t.catch = t.test(Catch)$statistic,
p.catch = t.test(Catch)$p.value,
mean.unilateral = mean(Unilateral),
mean.bilateral = mean(Bilateral),
mean.catch = mean(Catch)
)
}


##now we want t.tests for each load condition and group
##(but an anova within each group will be used in case of overall asymmetric indices)
## nan suggest no variability at all (e.g. catch trials in controls at ceiling except for auditory)
manyttestv0 = function(DF = Asymmetry) {
ddply(
DF,
c("Group", "Load"),
summarise,
df.unilateral = t.test(Unilateral)$parameter,
t.unilateral = t.test(Unilateral)$statistic,
p.unilateral = t.test(Unilateral)$p.value,
df.bilateral = t.test(Bilateral)$parameter,
t.bilateral = t.test(Bilateral)$statistic,
p.bilateral = t.test(Bilateral)$p.value,
df.catch = t.test(Catch)$parameter,
t.catch = t.test(Catch)$statistic,
p.catch = t.test(Catch)$p.value,
mean.unilateral = mean(Unilateral),
mean.bilateral = mean(Bilateral),
mean.catch = mean(Catch)
)
}


##preparing one ways anovas
OWanovas = function(whichGroup, whichType, DF = Asymmetry) {
colnames(DF)[colnames(DF) == whichType] = "dv"
DF = ddply(DF[DF$Group == whichGroup, ], c("ID", "Load"), summarise,
dv = mean(dv))
DF$ID = as.factor(DF$ID)
levels(DF$ID) = 1:length(levels(DF$ID))
return(ezANOVA(
DF,
dv = .(dv),
wid = .(ID),
within = .(Load),
detailed = T,
type = 3
))
}


##but ezanova does not provide partial eta square by default, here's the function:
extract.pes = function(EZ) {
return(cbind(
Effects = EZ$ANOVA$Effect,
pes = EZ$ANOVA$SSn / (EZ$ANOVA$SSn + EZ$ANOVA$SSd)
))
}


##posthoc t tests comparisons, one load condition vs another
#to be modified if design is not paired for some reason... can see no reasons
posthocOW = function(whichGroup,
whichType,
whichConditions,
DF = Asymmetry) {
colnames(DF)[colnames(DF) == whichType] = "dv"
DF = DF[DF$Group == whichGroup, ]
DF = DF[DF$Load %in% whichConditions, ]
return(t.test(
x = DF$dv[DF$Load == whichConditions[1]],
y = DF$dv[DF$Load == whichConditions[2]],
paired = T
))
}
```

So we can do:

```
OWanovas(whichGroup = "RHD", whichType = "Catch")
```

```
## $ANOVA
##        Effect DFn DFd         SSn        SSd         F         p p<.05
## 1 (Intercept)   1  11 0.002143347 0.07295953 0.3231492 0.5811406      
## 2        Load   2  22 0.010459534 0.12740055 0.9030956 0.4198174      
##          ges
## 1 0.01058425
## 2 0.04961366
## 
## $`Mauchly's Test for Sphericity`
##   Effect         W          p p<.05
## 2   Load 0.5576896 0.05394644      
## 
## $`Sphericity Corrections`
##   Effect      GGe     p[GG] p[GG]<.05       HFe     p[HF] p[HF]<.05
## 2   Load 0.693332 0.3904033           0.7614406 0.3981316
```

```
OWanovas(whichGroup = "RHD", whichType = "Unilateral")
```

```
## $ANOVA
##        Effect DFn DFd        SSn       SSd         F          p p<.05
## 1 (Intercept)   1  11 0.08522838 1.1083154 0.8458893 0.37744577      
## 2        Load   2  22 0.07002708 0.2963633 2.5991672 0.09698043      
##          ges
## 1 0.05720382
## 2 0.04748546
## 
## $`Mauchly's Test for Sphericity`
##   Effect        W         p p<.05
## 2   Load 0.649897 0.1159372      
## 
## $`Sphericity Corrections`
##   Effect       GGe     p[GG] p[GG]<.05       HFe     p[HF] p[HF]<.05
## 2   Load 0.7406842 0.1158189           0.8287127 0.1091045
```

```
OWanovas(whichGroup = "RHD", whichType = "Bilateral")
```

```
## $ANOVA
##        Effect DFn DFd       SSn       SSd        F           p p<.05
## 1 (Intercept)   1  11 0.4386537 3.1025840 1.555217 0.238266339      
## 2        Load   2  22 0.3821791 0.6875875 6.114087 0.007734431     *
##          ges
## 1 0.10372945
## 2 0.09159803
## 
## $`Mauchly's Test for Sphericity`
##   Effect         W          p p<.05
## 2   Load 0.5585213 0.05434989      
## 
## $`Sphericity Corrections`
##   Effect       GGe     p[GG] p[GG]<.05       HFe      p[HF] p[HF]<.05
## 2   Load 0.6937321 0.0180195         * 0.7620034 0.01490467         *
```

Are AI lateralized?

```
manyttestv0()
```

```
##     Group     Load df.unilateral t.unilateral p.unilateral df.bilateral
## 1     RHD   Visual            11  -1.43841245    0.1781549           11
## 2     RHD Auditory            11  -0.69527554    0.5013064           11
## 3     RHD   Single            11  -0.08854571    0.9310345           11
## 4 Control   Visual             6  -0.93419873    0.3862553            6
## 5 Control Auditory             6   0.00000000    1.0000000            6
## 6 Control   Single             6  -1.00000000    0.3559177            6
##     t.bilateral p.bilateral df.catch    t.catch   p.catch mean.unilateral
## 1 -2.527982e+00  0.02807403       11 -0.8043997 0.4382059     -0.10893246
## 2 -9.718228e-01  0.35202381       11  0.8206518 0.4292705     -0.03240741
## 3  4.488899e-02  0.96500033       11 -1.4832397 0.1660868     -0.00462963
## 4 -3.979662e-16  1.00000000        6 -1.0000000 0.3559177     -0.03174603
## 5 -1.000000e+00  0.35591768        6        NaN       NaN      0.00000000
## 6 -1.549193e+00  0.17230830        6        NaN       NaN     -0.01587302
##   mean.bilateral   mean.catch
## 1  -2.453704e-01 -0.027777778
## 2  -9.041394e-02  0.013888889
## 3   4.629630e-03 -0.009259259
## 4  -2.974102e-17 -0.007936508
## 5  -4.761905e-02  0.000000000
## 6  -1.587302e-02  0.000000000
```

```
posthocOW(whichGroup= "RHD", whichType= "Bilateral", 
          whichConditions= c("Single", "Auditory"))
```

```
## 
##  Paired t-test
## 
## data:  DF$dv[DF$Load == whichConditions[1]] and DF$dv[DF$Load == whichConditions[2]]
## t = 2.2362, df = 11, p-value = 0.04701
## alternative hypothesis: true difference in means is not equal to 0
## 95 percent confidence interval:
##  0.00149803 0.18858912
## sample estimates:
## mean of the differences 
##              0.09504357
```

```
posthocOW(whichGroup= "RHD", whichType= "Bilateral", 
          whichConditions= c("Single", "Visual"))
```

```
## 
##  Paired t-test
## 
## data:  DF$dv[DF$Load == whichConditions[1]] and DF$dv[DF$Load == whichConditions[2]]
## t = 2.8947, df = 11, p-value = 0.01458
## alternative hypothesis: true difference in means is not equal to 0
## 95 percent confidence interval:
##  0.05991275 0.44008725
## sample estimates:
## mean of the differences 
##                    0.25
```

```
posthocOW(whichGroup= "RHD", whichType= "Bilateral", 
          whichConditions= c("Visual", "Auditory"))
```

```
## 
##  Paired t-test
## 
## data:  DF$dv[DF$Load == whichConditions[1]] and DF$dv[DF$Load == whichConditions[2]]
## t = -1.9428, df = 11, p-value = 0.07807
## alternative hypothesis: true difference in means is not equal to 0
## 95 percent confidence interval:
##  -0.33050827  0.02059542
## sample estimates:
## mean of the differences 
##              -0.1549564
```

## Session

We repeat the mixed models procedure above. The first and last sessions were always performed with the single task. Also, controls are at ceiling, and were discarded.

```
data_mm= data[data$Load== "Single",]
data_mm$Session= factor(data_mm$Session)

#values
with(data_mm, 
     tapply(AccuracyPosition, list(Session, Group, Type), mean))
```

```
## , , Catch
## 
##         RHD Control
## 1 0.9814815       1
## 6 0.9814815       1
## 
## , , Left
## 
##         RHD  Control
## 1 0.9074074 0.984127
## 6 0.8981481 0.984127
## 
## , , Bilateral
## 
##         RHD  Control
## 1 0.7685185 0.952381
## 6 0.7685185 0.984127
## 
## , , Right
## 
##         RHD  Control
## 1 0.9166667 1.000000
## 6 0.8981481 0.968254
```

```
data_mm= data_mm[data_mm$Group== "RHD",] 
data_mm$Group= factor(data_mm$Group)
```

We select random effects:

```
#from here we select the random effects
mod0= glmer(AccuracyPosition ~ (1|Subject), 
            family = "binomial", data= data_mm)

mod0a= glmer(AccuracyPosition ~ (1+Session|Subject), 
            family = "binomial", data= data_mm)

anova(mod0, mod0a) #n.s.
```

```
## Data: data_mm
## Models:
## mod0: AccuracyPosition ~ (1 | Subject)
## mod0a: AccuracyPosition ~ (1 + Session | Subject)
##       Df    AIC    BIC  logLik deviance  Chisq Chi Df Pr(>Chisq)
## mod0   2 557.75 567.27 -276.87   553.75                         
## mod0a  4 559.75 578.79 -275.87   551.75 1.9996      2      0.368
```

```
mod0b= glmer(AccuracyPosition ~ (1+Type|Subject), 
             family = "binomial", data= data_mm)

anova(mod0, mod0b) #yes
```

```
## Data: data_mm
## Models:
## mod0: AccuracyPosition ~ (1 | Subject)
## mod0b: AccuracyPosition ~ (1 + Type | Subject)
##       Df    AIC    BIC  logLik deviance  Chisq Chi Df Pr(>Chisq)    
## mod0   2 557.75 567.27 -276.87   553.75                             
## mod0b 11 484.57 536.95 -231.29   462.57 91.171      9  9.476e-16 ***
## ---
## Signif. codes:  0 '***' 0.001 '**' 0.01 '*' 0.05 '.' 0.1 ' ' 1
```

```
#fixed effects
afex_mod= mixed(AccuracyPosition ~ Type*Session + (1+Type|Subject), 
                family = "binomial", data= data_mm, all_fit = T, 
                type = 2, method = "LRT")
```

```
## Fitting 5 (g)lmer() models:
## [.....]
```

```
afex_mod$anova_table
```

```
## Mixed Model Anova Table (Type 2 tests, LRT-method)
## 
## Model: AccuracyPosition ~ Type * Session + (1 + Type | Subject)
## Data: data_mm
## Df full model(s): 15
## Df full model(s): 15
## Df full model(s): 18
##              Df  Chisq Chi Df Pr(>Chisq)  
## Type         12 8.9529      3    0.02992 *
## Session      14 0.1497      1    0.69883  
## Type:Session 15 0.1951      3    0.97837  
## ---
## Signif. codes:  0 '***' 0.001 '**' 0.01 '*' 0.05 '.' 0.1 ' ' 1
```

No effects.

# Appendix

Package versions:

```
sessionInfo()
```

```
## R version 3.5.1 (2018-07-02)
## Platform: x86_64-w64-mingw32/x64 (64-bit)
## Running under: Windows 10 x64 (build 17134)
## 
## Matrix products: default
## 
## locale:
## [1] LC_COLLATE=Italian_Italy.1252  LC_CTYPE=Italian_Italy.1252   
## [3] LC_MONETARY=Italian_Italy.1252 LC_NUMERIC=C                  
## [5] LC_TIME=Italian_Italy.1252    
## 
## attached base packages:
## [1] stats     graphics  grDevices utils     datasets  methods   base     
## 
## other attached packages:
##  [1] bindrcpp_0.2.2 emmeans_1.3.2  gridExtra_2.3  dplyr_0.7.8   
##  [5] magrittr_1.5   afex_0.22-1    lme4_1.1-20    Matrix_1.2-14 
##  [9] plyr_1.8.4     ez_4.4-0       ggplot2_3.1.0 
## 
## loaded via a namespace (and not attached):
##  [1] jsonlite_1.6           splines_3.5.1          carData_3.0-2         
##  [4] gtools_3.8.1           assertthat_0.2.0       cellranger_1.1.0      
##  [7] yaml_2.2.0             numDeriv_2016.8-1      pillar_1.3.1          
## [10] lattice_0.20-35        glue_1.3.0             digest_0.6.18         
## [13] minqa_1.2.4            colorspace_1.4-0       sandwich_2.5-0        
## [16] htmltools_0.3.6        pkgconfig_2.0.2        haven_2.0.0           
## [19] BayesFactor_0.9.12-4.2 purrr_0.3.0            xtable_1.8-3          
## [22] mvtnorm_1.0-8          scales_1.0.0           openxlsx_4.1.0        
## [25] rio_0.5.16             MatrixModels_0.4-1     tibble_2.0.1          
## [28] mgcv_1.8-24            BayesFactorExtras_0.1  car_3.0-2             
## [31] TH.data_1.0-10         withr_2.1.2            pbapply_1.4-0         
## [34] lazyeval_0.2.1         cli_1.0.1              survival_2.42-3       
## [37] crayon_1.3.4           readxl_1.2.0           estimability_1.3      
## [40] evaluate_0.12          fansi_0.4.0            nlme_3.1-137          
## [43] MASS_7.3-50            forcats_0.3.0          foreign_0.8-70        
## [46] tools_3.5.1            data.table_1.12.0      hms_0.4.2             
## [49] multcomp_1.4-8         stringr_1.4.0          munsell_0.5.0         
## [52] zip_1.0.0              compiler_3.5.1         rlang_0.3.1           
## [55] grid_3.5.1             nloptr_1.2.1           labeling_0.3          
## [58] base64enc_0.1-3        rmarkdown_1.11         codetools_0.2-15      
## [61] gtable_0.2.0           lmerTest_3.1-0         abind_1.4-5           
## [64] curl_3.3               reshape2_1.4.3         R6_2.3.0              
## [67] zoo_1.8-4              knitr_1.21             utf8_1.1.4            
## [70] bindr_0.1.1            stringi_1.2.4          parallel_3.5.1        
## [73] Rcpp_1.0.0             tidyselect_0.2.5       xfun_0.4              
## [76] coda_0.19-2
```
